# Investigating the impact of feed-induced, subacute ruminal acidosis on rumen epimural transcriptome and metatranscriptome in young calves at 8- and 17-week of age

**DOI:** 10.3389/fvets.2024.1328539

**Published:** 2024-02-21

**Authors:** Wenli Li, Anna Larsen, Priscila Fregulia

**Affiliations:** ^1^US Dairy Forage Research Center, USDA-Agricultural Research Service, Madison, WI, United States; ^2^Oak Ridge Institute for Science and Education, Oak Ridge, TN, United States; ^3^Department of Animal and Dairy Sciences, University of Wisconsin-Madison, Madison, WI, United States

**Keywords:** feed-induced acidosis, young calves, rumen epithelial transcriptome, rumen epithelial etatranscriptome, microbial community changes

## Abstract

**Introduction:**

With the goal to maximize intake of high-fermentable diet needed to meet energy needs during weaning period, calves are at risk for ruminal acidosis. Using the calves from previously established model of feed-induced, ruminal acidosis in young calves, we aimed to investigate the changes in rumen epimural transcriptome and its microbial metatranscriptome at weaning (8-week) and post-weaning (17-week) in canulated (first occurred at 3 weeks of age) Holstein bull calves with feed-induced subacute ruminal acidosis.

**Methods:**

Eight bull calves were randomly assigned to acidosis-inducing diet (Treated, *n* = 4; pelleted, 42.7% starch, 15.1% neutral detergent fiber [NDF], and 57.8% nonfiber carbohydrates), while texturized starter was fed as a control (Control, *n* = 4; 35.3% starch, 25.3% NDF, and 48.1% nonfiber carbohydrates) starting at 1 week through 17 weeks. Calves fed acidosis-inducing diet showed significantly less (*p* < 0.01) body weight over the course of the experiment, in addition to lower ruminal pH (*p* < 0.01) compared to the control group. Rumen epithelial (RE) tissues were collected at both 8 weeks (via biopsy) and 17 weeks (via euthanasia) and followed for whole transcriptome RNA sequencing analysis. Differentially expressed genes (DEGs) analysis was done using cufflinks2 (fold-change ≥2 and *p* < 0.05) between treated and control groups at 8-week of age, and between 8- and 17-week for the treated group.

**Results:**

At 8-week of age, DEGs between treatment groups showed an enrichment of genes related to the response to lipopolysaccharide (LPS) (*p* < 0.005). The impact of prolonged, feed-induced acidosis was reflected by the decreased expression (*p* < 0.005) in genes involved in cell proliferation related pathways in the RE at 17-week of age in the treated group. Unique sets of discriminant microbial taxa were identified between 8-and 17-week calves in the treated group and the treatment groups at 8-week, indicating that active microbial community changes in the RE are an integral part of the ruminal acidosis development and progression.

## Highlights

- High-fermentable diet in young calves during weaning period induces ruminal acidosis.
- Prolonged feed-induced rumen acidosis affects transcriptome expression in the rumen epithelium.
- Microbial community changes in the rumen epithelium are an integral part of the ruminal acidosis development and progression.
- Response to lipopolysaccharide was a major response in the rumen epithelium at 8 weeks of age.

## Introduction

In the neonatal ruminants, the rumen is incompletely developed. As the calf consumes more starter feed, ruminal pH decreases while the short chain fatty acids (SCFAs), produced by ruminal fermentation, gradually increase. Intraruminal administration of SCFAs, such as acetate, propionate, and butyrate, can stimulate the growth of RE and rumen mass in young ruminants (1–3). Thus, the presence and absorption of SCFAs in the rumen is believed to be the required chemical stimuli for the proliferation of functional rumen papillae. However, highly processed, high-starch diets have been shown to induce acidosis in ruminants (4, 5). Calves fed a high-concentrate diet displayed excessive SCFA and lactic acid production due to rapid microbial breakdown of carbohydrates, which leads to significant decrease in rumen pH (6). An overall reduction in ruminal pH (7, 8) caused by ingestion of diets rich in rapidly fermentable carbohydrates with insufficient amount of fiber required for efficient rumen buffering can lead to subacute Ruminal Acidosis (SARA), a common metabolic disorder in dairy cattle. SARA is a well-recognized, economically important disorder in dairy cattle. In adult cattle, milk yield reduction, premature culling and increased mortality are among the direct consequences of SARA-induced digestive and metabolic disfunctions. However, long-term impact of consumption of highly fermentable diet from birth until beyond the weaning period is not well understood. SARA is characterized in general with low ruminal pH between 5.2 to 6 for prolonged periods (9, 10). However, studies have shown that there is variation in animal susceptibility to feed induced acidosis and the severity of the pH depression required to cause SARA symptoms varies among cows (11–13). Gene expression-based biomarkers have been investigated as predictive biomarker for disease detection (14–16). These biomarkers carry the high potential as an alternative to or to be used in conjunction with ruminal pH measurement for early diagnosis or prevention of acidosis.

We have established a model of feed-induced acidosis in young calves, where highly processed, fermentable diet was offered to calves at birth until 16 weeks of age to obtain a prolonged acidosis (17, 18). With this model, we observed significant difference in mean ruminal pH values between Treated and Control groups. Most importantly, greater degree of tissue degradation was observed in acidotic calves (*p* < 0.01) (18). The main objectives of this works are (1) to investigate the changes in RE transcriptome and the associated microbial community at weaning (8 weeks of age) between the treatment groups; and (2) to investigate the response of host RE to prolonged, feed-induced acidosis, through the comparative transcriptomics analysis in the RE between the two time points, 8 and 17 weeks of age, in the treated group. We hypothesize that feed-induced acidosis is accompanied with significant gene expression changes in the rumen epithelium in calves. Additionally, we hypothesize that significant shift in the microbiota associated to the rumen epithelium, the epimural microbiota, is an integral part of the host response to feed-induced acidosis, and that this response differs at weaning and post-weaning periods.

## Materials and methods

All animal protocols (A005848) were approved by the Animal Care and Use Committee at the University of Wisconsin–Madison. All the procedures relating to animal care and use in this study were implemented in accordance with the guidelines and regulations by the US Dairy Forage Research Center Farm. All the Holstein bull calves included in this study were from the same study published previously (17–20).

### Experimental design, measurements, and sample collection

This study is part of a larger study, where other portions of the study have been published. The procedure for feed induced acidosis was described previously (17–20). In brief, 8 Holstein bull calves were enrolled for this experiment. For the first 8 weeks after birth, calves were housed in individual calf hutches (4.8 m^2^/calf), then they were moved to divided super hutches (5.0 m^2^/calf), where the calves had their own individual pen, and could only access their own feed and water, through 16 weeks of age. Two diets were administered to the calves. One was a pelleted, low-fiber diet. This diet was designed to cause ruminal acidosis (Treated; pelleted, 42.7% starch, 15.1% neutral detergent fiber [NDF], and 57.8% nonfiber carbohydrates). Texturized starter was fed as a control diet (35.3% starch, 25.3% NDF, and 48.1% nonfiber carbohydrates). The feed is manufactured by Vita Plus Cooperation (Wisconsin, United States). Complete nutrient composition of each diet is provided in Gelsinger et al. (17), and the effects of the two diets were reported previously (17, 18). Eight Holstein bull calves were randomly assigned to an acidosis inducing diet (Treated; *n* = 4) and control diet (*n* = 4), via random number generator tool by graphpad.^1^ Calf starters were offered to calves at 1 week of age and lasted until 17 weeks, using bull calves born between June 17 and July 5, 2017. Calves had *ad libitum* access to water for the duration of the study.

At 3 weeks of age, soft rubber cannulas (28 mm i.d.) were surgically fitted to each calf following the method of Kristensen and coauthors (21). Between 7 and 9 weeks of age, larger soft rubber cannulas (51 mm i.d.; Bar-Diamond Inc., Parma, ID, United States) were used to replace the original cannulas to accommodate the increase in the size of the fistula. At 8 weeks, RE biopsy at the cranial sac was performed using a uterine biopsy tool for each calf. At 17 week, calves were euthanized by stunning using captive bolt for digestive tract tissue collection, including RE, liver, small intestine, and caecum. Rumen papillae tissues from the left and right sides of the cranial ventral sac were fixed for histology analysis to assess the lesion scores (18). At both time points, RE tissues were collected and rinsed in 1X PBS to remove the remaining feed particles. Cleaned tissues were immediately frozen in liquid nitrogen and stored at −80°C for further RNA extraction.

A calibrated pH electrode (Oakton, Cole Parmer Instrument, United States) was inserted into the rumen through the canula before each collection for 45 s to measure ruminal pH. Ruminal pH was measured at seven time-points (−8, −4, 0, 2, 4, 8, and 12 h relative to grain feeding) in a single day every other week from 6 to 16 week. Body weight was recorded weekly. A measured amount of starter was offered daily at 0800 h and refusals were determined daily. When refusal <200 g was recorded on 2 consecutive days, A calf’s daily allotment of starter was increased (18). Health scores of the calves were monitored by measuring heart and respiratory rates, rectal temperature, fecal consistency, and naval characteristics on weekly basis.

### RNA sequencing power analysis

Using the Bioconductor Package ssizeRNA (22), we determined that 4 biological replicates per treatment with 20 million reads per sample would be sufficient to identify differentially expressed genes from RNA-sequencing data at a power of 0.90 using the following parameters: statistical power cutoff = 0.8, number of genes = 20,000, minimum number of DEGs = 200, average read count = 1,000, and fold change = 2, FDR = 0.05. We obtained 50–60 million reads per sample in our experiment design. Thus, the sequencing depth would ensure sufficient statistical power for transcriptome analysis.

### RNA extraction and sequencing library preparation

For host total RNA extraction, 50 mg rumen papillae tissues were homogenized on the Precellys homogenizer (Bertin Instrument, France) at 7,500 RPM for 30 s per cycle, with 4 repeats. The tissue homogenate was place in ice for 1 min between cycles. After homogenization, total RNAs were extracted from both tissue types following the miRNeasy protocol with a QIAcube instrument (Qiagen, United States). The quality of extracted RNAs was assessed using Bioanalyzer RNA 6000 nano kit (Agilent Technologies, United States). RNA samples with RNA integrity number (RIN) value ≥8 were pursued for RNA quantification using Qubit (Thermo Fisher, United States). RNA-sequencing library preparation was done using Illumina TruSeq ribo-zero Gold kit following manufacturer’s instructions. For each sample, 1 *μg* of total RNA was used for sequencing library preparation. Quantification of prepared libraries was performed using a Kapa quantification kit following manufacturer’s instruction (KK4873, Kapa systems, Roche, Switzerland) with a QuanStudio 5 RT-qPCR instrument (ThermoFisher Scientific, United States). Using the concentration generated by Kapa kit, sequencing pooling was prepared according to the calculation offered by the pooling calculator.^2^ Pooled libraries were initially sequenced using an Illumina MiSeq nano 300-cycle kit. The pooling was normalized further to ensure equal depth of sequencing of all the libraries, according to the index ratios generated by the nano kit run. The finally normalized, pooled libraries were sequenced on the Illumina NextSeq 500 instrument, using a high-output 300-cycle cartridge to generate paired-end, 2x150bp reads.

### Bioinformatics analysis of RE sequencing reads and taxa classification of microbial reads

Sequencing data analysis followed the procedure described in the co-author’s publication (20). RNA sequencing raw data for the RE tissues at 8 weeks of age were generated from this study, the ones at 17 week of age were obtained from our previously published work (20). Briefly, raw reads were mapped to the cattle reference genome (ARS-UCD 1.2) using STAR (2.5.2b) (23). Differentially expressed gene (DEG) analysis was performed by the cuffdiff package in cufflinks2 (24). To determine significantly, differentially expressed genes, these cutoff values were used: FPKM ≥5, adjust-value of *p* <0.05 and fold-change (FC) ≥2. Gene ontology (GO) and pathway analysis were performed using DAVID (25). DEGs analysis and associated GO analysis were done for these comparisons: between the treatment groups at 8 week of age, and between the 8 week and 17 week of age in the treatment group. Additionally, DEGs between the 8 and 17 week in calves fed control diet were obtained to identify differentially expressed genes due to growth and maturity. And these DEGs were filtered out from the list of DEGs between the two time points in the treated group to identify DEGs due to prolonged acidosis.

Unmapped reads to the cattle reference genome were considered of microbial origin. To further enrich microbial rRNA reads, SortMeRNA (version, 2.1b) (26) was used to map host genome unmapped reads to the reference rRNA databases provided by SILVA (release 138) (27) and Rfam 11.0 (28). The enriched rRNA reads were used for bacterial taxonomic classification, by Kraken2 (v.2.0.8-beta) (29) using the standard database that includes bacteria and Archaea. Raw-read counts at each taxonomic level (i.e., phylum and genus) as identified by Kraken2 were normalized by total number of classified reads per sample by these steps: (1) the total number of reads mapped to the given taxonomic level (i.e., phylum, genus, and species) was divided by 1,000,000 to obtain the “per million factor”; (2) the total number of reads mapped to each specific given taxonomic level was divided by the “per million factor” to yield the normalized read count. Normalized read counts at a given taxonomic level was used as the measurement for microbial abundance analysis. The normalized read count of 2 or more was used to determine the presence/expression of taxa. Differential abundance analysis of microbial taxa at genus level was done using DEseq2 (with Benjamini and Hochberg method for multiple testing) using the raw read counts mapped to the given taxa (*p* < 0.05; mean read count ≥10 and fold-change ≥2). For the RE tissues collected at 17 weeks of age, we used genus-level reads counts from our previously published work (19). It is known that rumen development is accompanied by microbial colonization (30). We were interested in identifying microbial signature changes in the RE due to prolonged, ruminal acidosis by comparing the microbial communities in the treated calves between 8 and 17 weeks. Thus, microbial genera that showed significant differential abundance changes due to development and maturation between the two timepoints in the control group were removed (44 of them) from the analysis.

### Analysis of microbial community and experimental variables

The mixMC multivariate method implemented in the *mixOmics* R package (31) was used to identify specific associations between microbial taxa abundance and experimental variables. We identified microbial signatures related to the following treatments: (1) treated vs. control for animals at 8 week of age; (2) samples collected at 8 and 17 week of age for the treated group, and 17 week for the control group. Microbial taxa that showed significant abundance differences between the two time points (8 and 17 week) in the control group were considered being affected by growth and were removed (44 of them) from the microbial analysis for both 8- and 17-week samples in the treated group. Within the statistical framework of the mixMC package, the sparse partial least square discriminant analysis (sPLS-DA) (32) was used to perform feature selection by sparsity assumption, presuming that a small number of features can drive a biological event (33). The microbial taxa with relative abundance >0.01% were included in this analysis. The optimal number of components was selected based on the average balanced classification error rate with maximum distance over 10 repeats of a 3-fold cross-validation of an sPLS-DA model. The optimal number of variables for each component was chosen based on the lowest average balanced classification error rate. Discriminant genera were plotted according to their contribution to component 1 of 2 of sPLS-DA.

## Results

### Ruminal pH, body weight and ruminal papillae degradation in the Treated and control groups

Detailed results for body weight, feed intake, ruminal pH and ruminal papillae were reported before (18, 20). Briefly, mean ruminal pH, which was 5.37 ± 0.24 (3.3, 7.2) and 5.63 ± 0.24 (3.5, 6.8) for Treated and control calves, respectively, differed by diet (*p* < 0.01). Body weight and feed intake showed linear increase with age (*p* < 0.01). At both 4 and 5 weeks of age, calves fed the control diet attained greater body weight (*p* < 0.01) and consumed greater amount of feed (*p* < 0.01) compared to the Treated group. These differences sustained through week 16 (*p* < 0.001). For ruminal papillae histology analysis, a greater degree of tissue degradation was observed for the Treated group compared to the control (*p* < 0.01) (18).

### Sequencing reads alignment for rumen epithelial tissues

For rumen tissues collected at 8 weeks of age, the average, total number of reads is 59.8 M ± 1.2 M, with an average mapping rate to the cattle reference as 76% ± 2%. For this time point, the average number of rRNA reads mapped to the rRNA database and used for microbial classification is 5.7 M ± 0.6 M (mean ± s.e.). Similarly, a high Kraken classification rate was obtained for these samples 99.2 ± 0.12 (mean ± s.e.). For rumen tissues collected at 17 weeks of age, the average, total number of reads is 57.5 M ± 0.4 M, with an average mapping rate to the cattle reference genome as 56.8% ± 4.3%. And for this time point, the average number of rRNA reads mapped to the rRNA database and used for rumen wall microbial classification is 9.3 M ± 1.6 M (mean ± s.e.). A high classification rate was achieved for each sample, with the mean Kraken classification rate as 99.39 ± 0.13 (mean ± s.e.).

### Gene expression profile in the RE at 8 weeks

A total of 82 genes showed significant differential expression changes between the treatment groups at 8 weeks of age (FPKM ≥5, FC ≥ 2 and value of *p* ≤0.05). Compared to the control group, 24 genes showed increase in expression and 58 of them showed decrease in expression in the treated group. GO analysis using identified DEGs indicated that these genes were enriched in the immune response related pathways. They include innate immune response (GO:0045087, 10 genes, value of *p* < 0.01), cellular response to lipopolysaccharide (LPS; GO:0071222, 5 genes, value of *p* < 0.05), and chemokine-medicated signaling pathway (GO:0070098, 4 genes, value of *p* < 0.05).

For the top 10 most highly expressed genes in the treated group, we observed a significant enrichment in the cellular pathways related to the stress response (*ISG15*, *S100A9*, *LGALS4*, *PPIF* and *PLIN2*). Specifically, 3 of these genes were identified with annotated function in response to bacteria (*ISG15*, *S100A9* and *LGALS4*).

### Rumen gene expression changes in the treated group between 17 and 8 weeks

For the treated group, we compared the gene expression profiles between the 8- and 17-week timepoints. Between the two time-points in the treated group, a total of 502 genes showed significant differential expression (FPKM ≥5, FC ≥ 2 and value of *p* ≤0.05). In the control group, a total of 313 DEGs were identified between 8 and 17 week. In the absence of acidosis inducing diet, these DEGs represented transcriptome changes due to development, were removed from the list of DEGs identified in the treated group. After the filtering, 357 DEGs were unique to the comparison in treated group and were followed for further analysis. For these genes, 112 of them were up-regulated and 245 of them were down-regulated at 17 week of age compared to 8 week of age. GO term analysis using the up-regulated genes at 17 week of age indicated predominant enrichments in cell proliferation and migration related pathways, and: extracellular space (GO:0005615, 24 genes, value of *p* << 0.0001), cell adhesion (GO:0007155, 24 genes, *p* << 0.0001), cell proliferation (GO:0008283, 24 genes, value of *p* << 0.0001), cell migration (GO:0016477, 19 genes; value of *p* << 0.0001), regulation of immune system process (GO:0002682, 20 genes, value of *p* < 0.01), lysosome (GO:0005763, 11 genes, value of *p* < 0.005), regulation of leukocyte activation (GO:0002694, 13 genes, value of *p* < 0.005), regulation of lymphocyte activation (GO:0051249, 12 genes, value of *p* < 0.005) and MHC class II protein complex (GO:0042613, 4 genes, value of *p* < 0.05; Table 1). GO term analysis using the down-regulated genes at 17 week of age indicated enrichment in lipid and organic acid metabolic processes. They include lipid metabolic process (GO:0006629, 24 genes, value of *p* < 0.00001), cholesterol biosynthesis (GO:0006695; 8 genes, value of *p* < 0.0001), organic acid metabolic process (GO:0006082, 31 genes, value of *p* < 0.05), carboxylic acid biosynthetic process (GO:0046394; 31 genes, value of *p* < 0.001) and organic hydroxy compound metabolic process (GO:1901615, 19 genes, value of *p* < 0.01; Table 2).

**Table 1:** The GO terms and associated genes identified using up-regulated genes at 17 week of age compared to the 8 week in treated group.

GO terms	Genes	Adjusted value of *p*
Extracellular space (GO:0005615; 24 genes)	*AXL, APOD, CTSK, CTSS, CSF1, ENPP2, EDN1, EDN3, FGL2, FBLN1, GPX3, IGFBP3, LPO, BoLA, MMP2, MFAP4, RBP4, RNASE6, SFRP1, SPARC, SERPINB1, SMPDL3B, TTR, XDH*	<<0.0001
Cell adhesion (GO:0007155; 24 genes)	*ANTXR1, AXL, EPHB6, XG, APOD, CLDN23, CLDN5, CSF1, DPT, DUSP3, EMILIN2, EFS, ECM2, FGL2, FBLN1, GPNMB, BOLA-DMA, BOLA-DMB, BOLA-DRA, BOLA-DRB3, MXRA8, MFAP4, NPNT, SFRP1*	<<0.0001
Cell proliferation (GO:0008283; 24 genes)	*EGFL7, EPHB6, KLF11, ATF5, APOD, CLDN5, CSF1, DPT, EDN1, FBLN1, GPNMB, IGFBP3, KRT4, MMP2, NUPR1, PDGFRB, SFRP1, SPARC, SERPINB1, SIRT1, SPHK1, TLR4, TYROBP, XDH*	<<0.0001
Cell migration (GO:0016477; 19 genes)	*AXL, XG, APOD, CLDN5, CSF1, ENPP2, EMILIN2, EFS, EDN1, GPNMB, IGFBP3, NTRK2, PLVAP, PDGFRB, SFRP1, SPARC, SIRT1, SPHK1, TLR4*	<<0.0001
Regulation of immune system process (GO:0002682; 20 genes)	*AXL, EPHB6, XG, APOD, CSF1, DUSP3, FGL2, GPNMB, BOLA-DMA, BOLA-DMB, BOLA-DRA, BOLA-DRB3, MFAP4, PLVAP, SFRP1, SIRT1, SMPDL3B, SPHK1, TLR4, TYROBP*	<0.01
Lysosome (GO:0005764; 11 genes)	*ARSA, CTSK, CTSS, FNIP2, BOLA-DMA, BOLA-DMB, BOLA-DRA, BOLA-DRB3, PDGFRB, RNASE6, SERPINB1*	<0.005
Regulation of leukocyte activation (GO:0002694; 13 genes)	*AXL, EPHB6, DUSP3, FGL2, GPNMB, BOLA-DMA, BOLA-DMB, BOLA-DRA, BOLA-DRB3, SFRP1, SPHK1, TLR4, TYROBP*	<0.005
Regulation of lymphocyte activation (GO:0051249; 12 genes)	*AXL, EPHB6, DUSP3, FGL2, GPNMB, BOLA-DMA, BOLA-DMB, BOLA-DRA, BOLA-DRB3, SFRP1, TLR4, TYROBP*	<0.005
MHC class II protein complex (GO:0042613; 4 genes)	*BOLA-DMA, BOLA-DMB, BOLA-DRA, BOLA-DRB3*	<0.05

**Table 2:** The GO terms and associated genes identified using down-regulated genes at 17 week of age compared to the 8 week in treated group.

GO terms	Genes	Adjusted value of *p*
Lipid metabolic process (GO:0006629; 24 genes)	*HMGCS1, HMGCS2, ST6GALNAC6, ACAT1, ACADVL, ACSF2, ALOX15B, CYP51A1, CYB5R1, FDPS, FDFT1, GSTM1, GPX4, GDE1, HSD17B12, ISYNA1, INSIG1, LSS, LIPN, MVD, PRDX6, SPTSSB, SCD, TECR*	<<0.00001
Cholesterol biosynthesis (GO:0006695; 8 genes)	*HMGCS1, HMGCS2, CYP51A1, FDPS, FDFT1, INSIG1, LSS, MVD*	<0.005
Organic acid metabolic process (GO:0006082; 31 genes)	*ACAT1, ACAT2, ACADVL, ACSF2, ACOT8, ALDOC, ACY1, ALOX15B, ENO1, FBP1, GGH, GGT1, GOT2, GSTM1, GSTA1, GPX4, HSD17B12, HSD17B8, INSIG1, IDH3A, MDH1, ME2, MTHFD1, ODC1, PGD, PSAT1, PSPH, PYCR1, SHMT2, SCD, TECR*	<0.05
Carboxylic acid biosynthetic process (GO:0046394; 31 genes)	*ACAT1, ACAT2, ACADVL, ACSF2, ACOT8, ALDOC, ACY1, ALOX15B, ENO1, FBP1, GGH, GGT1, GOT2, GSTM1, GSTA1, GPX4, HSD17B12, HSD17B8, INSIG1, IDH3A, MDH1, ME2, MTHFD1, ODC1, PGD, PSAT1, PSPH, PYCR1, SHMT2, SCD, TECR*	<0.001
Organic hydroxy compound metabolic process (GO:1901615; 19 genes)	*HMGCS1, HMGCS2, WNT5A, ALOX15B, CLCN2, CYP51A1, CYB5R1, FDPS, FDFT1, GDE1, ISYNA1, INSIG1, LSS, MSMO1, MVD, PGP, PDXP, SPTSSB, SQLE*	<0.01

Finally, for the up-regulated genes in the treated group (URGT) at both 8 and 17 week of age, we observed a total of 10 genes with significantly increased expression at both time points in the treated group. These genes include *CNFN*, *EPHB6*, *KLK12*, *KRTDAP*, *NOXA1*, *PI3*, *PSORS1C2*, *RAB38*, *S100A9* and *TMEM229B*. Pathway enrichment analysis indicated that these genes were enriched in cell membrane (GO:0016020; 4 genes, value of *p* < 0.05). For both timepoints, 8 genes showed significant expression decrease in the treated group. They included *BEST2*, *CCL20*, *CMPK2*, *ENTPD5*, *LGALS4*, *PRSS35*, *RBP4* and *UGT2B10*.

### Microbial community profile in the RE at 8 and 17 week of age

**Figure 1:**
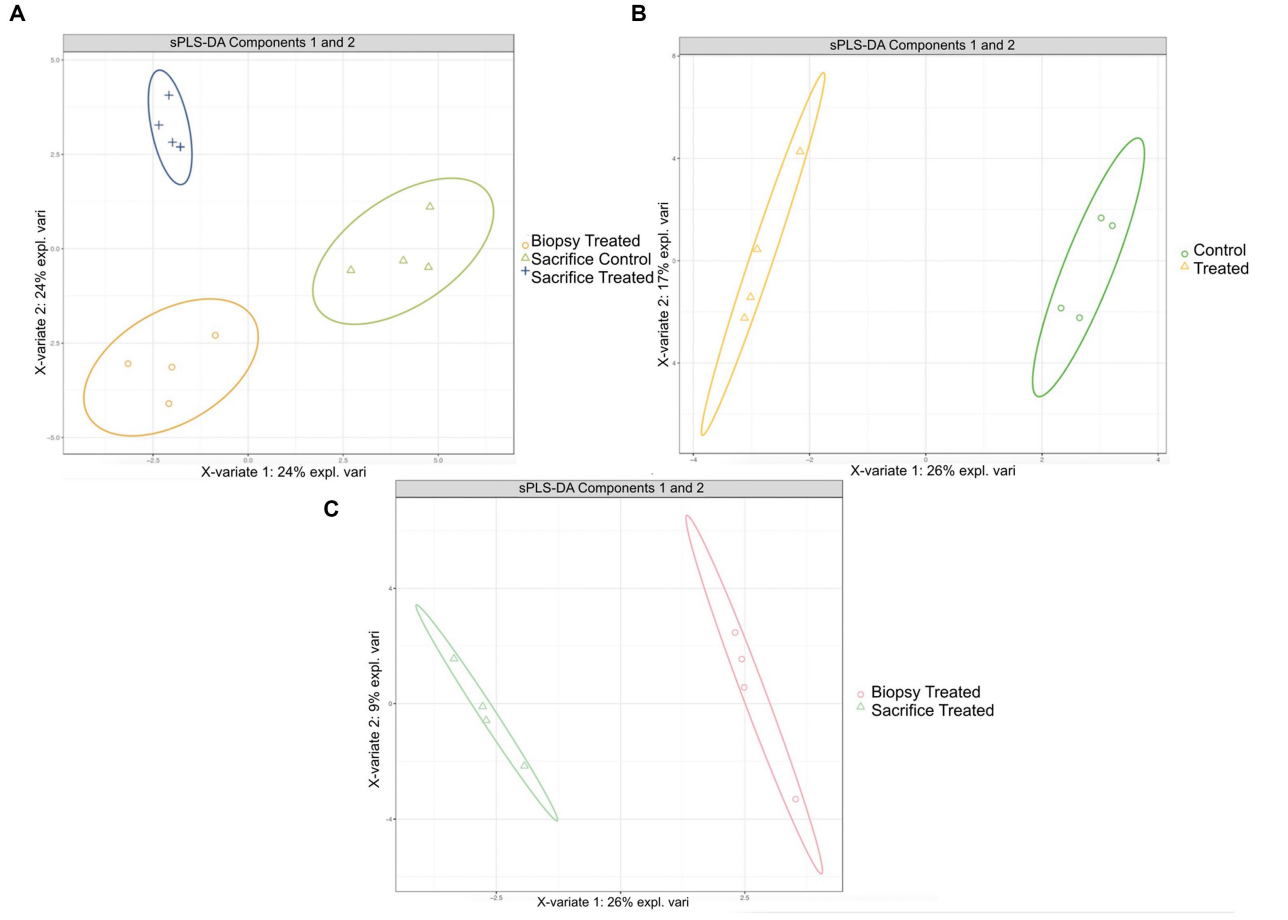
Results of sPLS-DA for microbial profile at genus level in rumen of calves. Individual score plot of the samples along the first two components, with 95% confidence level. (A) Microbial profiles for RE in treated calves at 8 weeks, treated and control calves at sacrifice (17 weeks). (B) Microbial profiles for RE samples collected from control and treated calves at 8 weeks; (C) Microbial profiles for RE samples collected at 8 weeks and 17 weeks for the treated calves.

**Figure 2:**
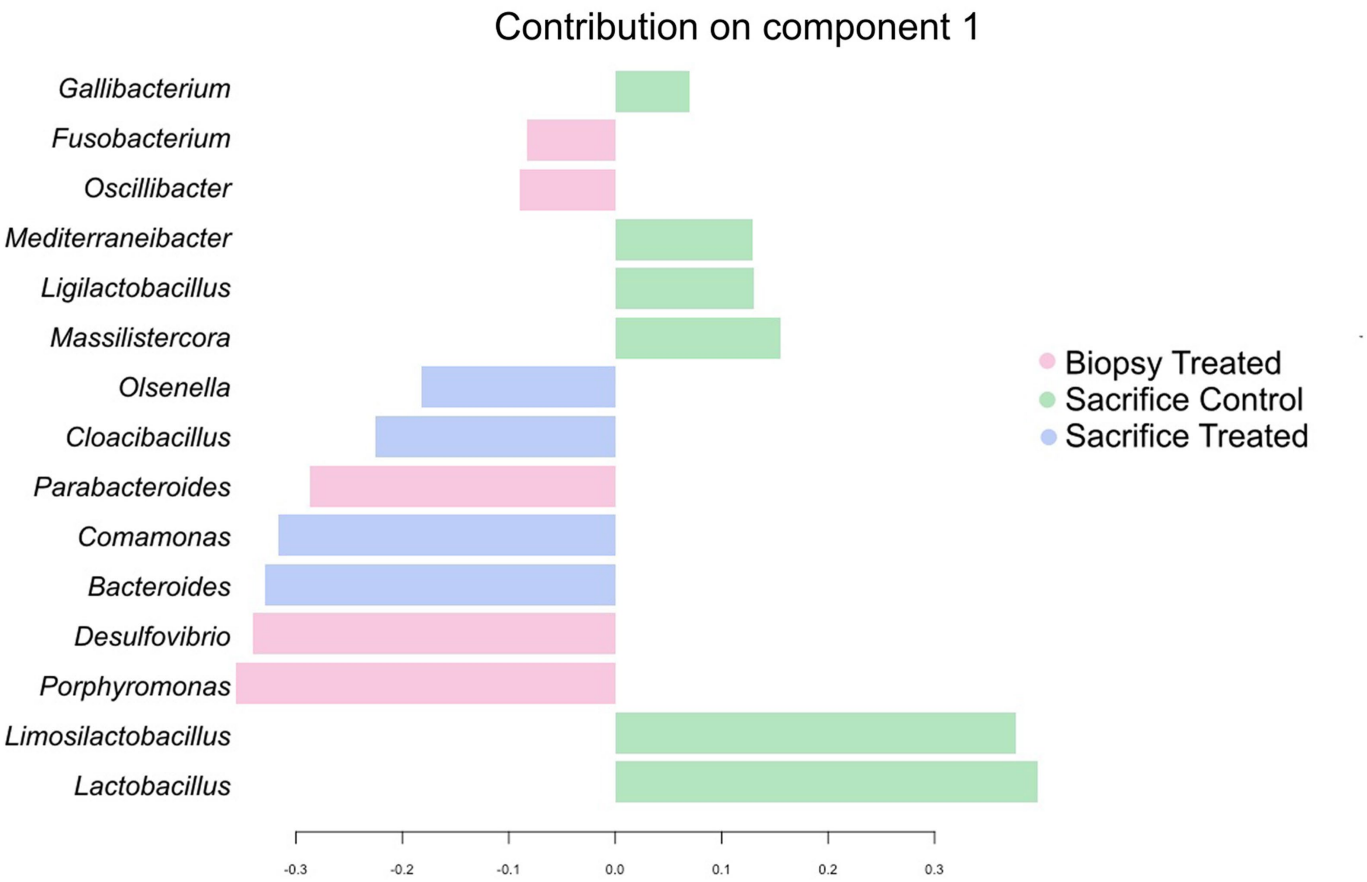
Genera contribution ranked from bottom (most important) to top for the Treated and Control groups collected at 8 week of age. Colors indicate the treatment in which the feature was most relevant.

**Figure 3:**
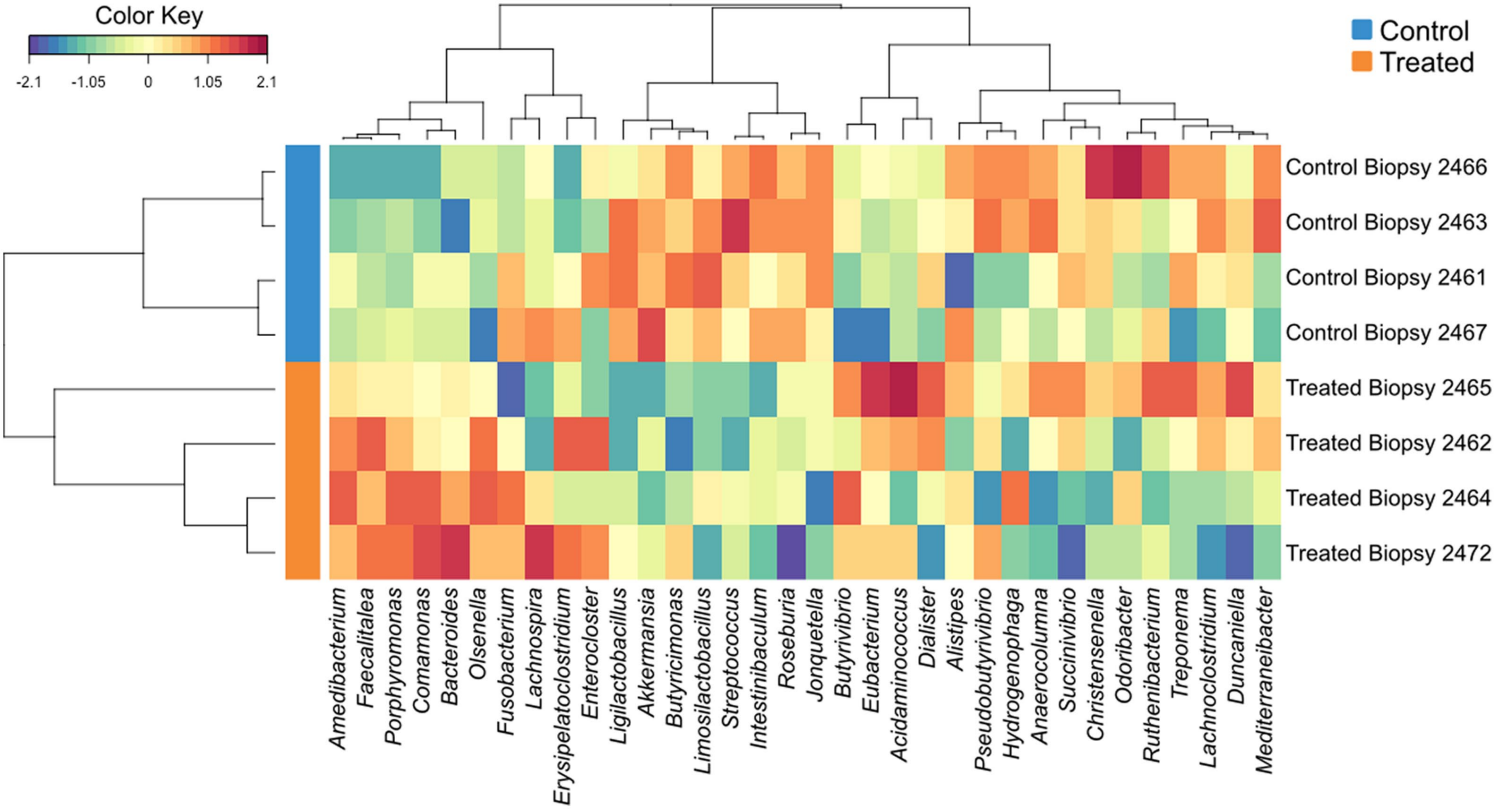
Hierarchical clustering (Euclidean distance, Ward linkage) of the selected genera from sPLS-DA results. The control groups were indicated by the blue bars, and treated groups were indicated by orange bars.

sPLS-DA analysis revealed a clear separation in the microbial profiles between the biopsy of treated animals (8 weeks), treated animals at sacrifice (17 weeks), and control animals at sacrifice (Figure 1A). Additionally, clear separation was identified for the following pairwise comparisons: control and treated samples collected at 8 weeks of age (Figure 1B); samples collected at 8 and 17 weeks for the treated animals (Figure 1C). Overall, 40% of the bacterial signature selected in component 1 of the sPLS-DA characterized the rumen microbiome of animals from the control group at sacrifice, which included the bacterial taxa *Gallibacterium*, *Mediterraneibacter*, *Ligilactobacillus*, *Massilistercora*, *Limosilactobacillus*, and L*actobacillus*. On the other hand, 33% of the microbial signature characterized the rumen microbiome of animals from the treated group at biopsy, and the signature of this group comprised the *Fusobacterium*, *Oscillibacter*, *Parabacteroides*, *Desultovibrio* and *Porphyromonas*. For the treated group at sacrifice, the microbial signature comprised of the *Olsenella*, *Cloacibacillus*, *Comamonas*, and *Bacteroides* (Figure 2). In accordance with the sPLS-DA analysis, the heatmap showed a clear distinction between the treatments (Figure 3).

## Discussion

### Host RE response to SARA induction at 8 week of age

In this experiment, we monitored the health scores of the calf by measuring heart and respiratory rates, rectal temperature, fecal consistency, and naval characteristics on weekly basis. As expected, we did not detect any qualitative difference in these measurements between the treatment groups at 8 week of age (unpublished data), since SARA is a subclinical disorder and clinical signs are not commonly observed. Our study indicated that the host’s response to the acidotic diet was captured by the transcriptome changes in the ruminal epithelial. Notably, for the DEGs, we observed a GO enrichment in the cellular response to lipopolysaccharide at the 8 week of age. Though cannulated animals on this study, which can potentially influence the rumen fermentation and inflammatory response (34), all the calves included in the comparative analysis were canulated, eliminating the difference due to cannulation alone. In this study, the treatment diet is a high-concentrate diet, aimed at inducing subacute ruminal acidosis. In cattle, diet-induced SARA models have resulted in deleterious changes in ruminal digesta and peripheral blood (35). These include ruminal pH depression, increased concentration of bacterial endotoxins [i.e., lipopolysaccharide (LPS)], and bioamines (e.g., histamine, tyramine and tryptamine). Moderate SARA has been reported to reduce ruminal gut barrier function, resulting in the translocation of these bacterial toxins (36, 37). Ruminal bacteria are predominantly Gram negative (38), which contain LPS in their thin peptidoglycan cell wall. LPS is important for the structural and functional integrity of the bacteria (39). Thus, Gram negative bacteria are the major source of LPS in the rumen. Significant increase (4- to 16-folder higher) of LPS concentration in the rumen has been reported in cows with SARA compared to the ones without SARA (40, 41). LPS can also translocate from the compromised RE barrier function into the blood stream and other parts of the body, invoking local and systemic inflammation response (36, 37, 42), by stimulating the release of proinflammatory cytokines, such as TNF-α, IL-1β, and IL-6 (43). Additionally, increased acute phase proteins, such as serum amyloid A, haptoglobin, C-reactive protein, and alpha-1-acid glycoprotein have been reported in cattle peripheral blood (40, 44).

Consistently with this, the most highly expressed genes in the treated group included 5 genes enriched in the cellular pathway of response to stress (*ISG15*, *S100A9*, *LGALS4*, *PPIF* and *PLIN2*). And 3 of these genes were identified with reported function in direct response to bacteria (*ISG15*, *S100A9* and *LGALS4*). Most notably, *PPIF* was reported with a role in regulating tissue necrosis due to stress or illness (45). The expression of these genes has not been studied before for their potential diagnostic power to detect with diet-induced SARA in cattle. Diet-based management of ruminal acidosis is highly desirable since it is relatively easy to manage and cost-effective on a farm without the devasting cost of clinical interventions. Further follow-up on these genes in a larger cohort may help determine if increased expression in the circulatory system in any of these genes may serve as effective early detection biomarkers for liability to develop ruminal acidosis.

### Host RE response to prolonged subacute acidosis at 17 week of age in the treated group

In our experiment, the effects of prolonged feeding of high-concentrate diet were captured by the changes in the transcriptome between 17- and 8-week-old calves fed acidosis inducing diet. We observed the trend of increased gene expression in genes related to cell proliferation, indicating that the prolonged, feed-induced acidosis may impact the rumen physical mass development. The rumen is the largest chamber in cattle’s stomach and it is the main location where the non-digestible feed is fermented and converted to absorbable nutrient by the cattle. Nutrients, like minerals and SCFAs, are absorbed by the rumen wall (46). Papillae protruding from the RE wall greatly increases the surface area for SCFA absorption that accounts for 75% of the metabolizable energy supply (47). Rumen papillae length, width and density directly contribute to the thickness of the RE, and their association with cattle feed efficiency has been reported (48, 49). As an adaptive response to increased SCFA levels due to high concentrate diet, increased size of the papillae was reported in sheep to maximize the surface area for SCFA absorption (50). It was reported in sheep and cattle that increased SCFA concentration level in the rumen can lead to rapid proliferation of stratified squamous rumen epithelium and its morphogenesis (3, 51).

Genes encoding lysosome and associated with immune process showed increase in expression at 17 week of age. Lysosomes are membrane-bound organelles with roles in processes involved in degrading and recycling cellular waste and dead cells, cellular signaling and energy metabolism. As an inherent part of the intracellular defense system against microbes, lysosome biogenesis is triggered by intracellular pathogen infections, and it plays a key role in pathogen detection and signaling (52). Thus, lysosome activities are directly related to host innate immunity (53–55). Yoon and co-authors (56) reported that lysosomes isolated from egg white displayed significant antimicrobial effect against several microbial organisms, suggesting that lysosomes might be a promising antibiotic alternative. Rumen epithelial erosion was observed with ruminal acidosis (57, 58). In our study, a greater scale of tissue degradation was observed in rumen papillae tissues in acidotic calves (18). With a prolonged low ruminal pH, the treated calves in our study did not show any clinical signs of SARA (unpublished data). We hypothesize that much of this is due to the innate immunity regulation of the host and its ability to fight off potential pathogens in close contact with the ruminal epithelium.

For down-regulated genes at 17 week of age, we observed enrichment in lipid metabolism and biosynthesis related pathways. In a healthy rumen, ruminal lipids derived from plants and microbes are absorbed across the RE and utilized for cell growth and proliferation by the epithelial cells (59). Thus, an important function of RE is lipid metabolism as suggested by Zhao and coauthors (60). In a study on transcriptome changes in lambs fed high-concentrate diet and normal diet, Sun and co-authors observed significant expression changes in lipid metabolism associated genes (61), suggesting that the absorptive capacity of ruminal lipids through the RE is critical in maintaining ruminal homeostasis. Steel and coauthors observed expression reduction in genes associated with cholesterol biosynthesis in both dry and lactating cows (58, 62). SCFAs are precursors of cholesterol. The concentration increase of SCFAs in acidotic calves means that the substrates available for cholesterol synthesis escalates. Thus, the downregulation of genes related to cholesterol biosynthesis might be an effective way to maintain the homeostasis of cholesterol. So far, we still have limited knowledge about the lipid metabolism capacity of the ruminal epithelium in the context of high-concentrate diet and its contribution to rumen development and maturity in young calves. Functional follow up of the key genes identified in this study that are involved in lipid metabolism and cholesterol biosynthesis may provide new insights in calves with feed induced SARA.

### RE microbial community changes with prolonged acidosis inducing diet treatment

Though high-concentrate diet has been considered the root cause of ruminal acidosis in dairy cattle, the cows may display differed phenotypic response and varied severity of ruminal acidosis to the feed treatment. The gut microbiological changes were considered essential in modulating systematic health in cattle. The RE microbiome is at the direct interface of rumen contents and the RE. Thus, it is expected that the RE microbiome may play an indispensable role in protecting the host from the intrusion of harmful microbes by forming a protective biofilm. However, the functional role of RE microbiome is not well understood. Previous studies have reported significant rumen microbial community changes during SARA through rumen content sequencing (63–65). In the experiment by Wetzels and co-authors (13), where a long-term, continuous SARA-inducing feeding challenge was performed, the cows were grouped as responders and non-responders. In agreement with our results, Petri et al. (66) found a high abundance of the genera *Olsenella* and *Comamonas* in animals with induced ruminal acidosis. The *Fusobacterium* genus is a normal inhabitant of the rumen of cattle, however, some species from this genus are opportunistic pathogens and primary causative of rumen abscesses (67, 68). The presence of this microbial genera in the treated animals highlights the possibility of using microbial taxa as biomarkers for subacute acidosis. Diet-specific, strong shifts in the highly abundant members of the rumen epimural communities were observed. In this study, our analysis identified unique set of microbial taxa that were differently correlated to the RE samples to the two time points in the treated group. These differences are expected considering the microbial colonization the rumen development. Furthermore, it suggests that the microbial communities in the RE are evolving, potentially as a mechanism for the host to defend any pathogen invasion at the time when the RE is most vulnerable.

## Conclusion

Using a feed-induced acidosis model in young calves, our whole transcriptome sequencing analysis indicated that the host response to acidosis-inducing diet can be captured as early as 8 week of age. At this time point, cellular response to lipopolysaccharide is the major response in the rumen epithelium. In the treated group fed an acidosis-inducing diet, 5 genes were identified with the highest expression at 8 weeks of age. These genes have reported role in direct response to bacteria. Unique set of microbial taxa were identified as the key taxa differing between the treatment groups at 8 weeks and between the two 8 and 17 week of age in the treated group. Our findings suggested that microbial community changes in the rumen epithelial is an integral and evolving part of the host’s response to feed-induced acidosis. Future follow up on the new genes and the key taxa identified in this study may facilitate the development of new biomarkers for the early diagnosis and prevention of feed-induced acidosis.

## Data availability statement

The datasets presented in this study can be found in online repositories. The names of the repository/repositories and accession number(s) can be found at: https://www.ncbi.nlm.nih.gov/, PRJNA948013, PRJNA1025110.

## Ethics statement

The animal study was approved by IACUC committee of University of Wisconsin-Madison. The study was conducted in accordance with the local legislation and institutional requirements.

## Author contributions

WL: Conceptualization, Data curation, Formal analysis, Investigation, Methodology, Supervision, Writing – original draft, Writing – review & editing. AL: Methodology, Writing – review & editing. PF: Formal analysis, Methodology, Writing – review & editing.

## Footnotes

^1^ https://www.graphpad.com/quickcalcs/randomize1/

^2^ https://support.illumina.com/help/pooling-calculator/pooling-calculator.htm
